# Vanishing Adrenal Glands: Bilateral Adrenal Hemorrhage With Adrenal Insufficiency in COVID-19 Infection

**DOI:** 10.7759/cureus.41210

**Published:** 2023-06-30

**Authors:** Priyanka Majety, Natanie Erlikh, Runhua Hou

**Affiliations:** 1 Endocrinology, Diabetes, and Metabolism, Virginia Commonwealth University, Richmond, USA; 2 Endocrinology, Diabetes, and Metabolism, Beth Israel Deaconess Medical Center, Harvard Medical School, Boston, USA; 3 Endocrinology, Diabetes, and Metabolism, Massachusetts General Hospital, Harvard Medical School, Boston, USA

**Keywords:** coronavirus disease 2019, mineralocorticoid, antiphospholipid syndrome, covid-19 infection, adrenal insufficiency, adrenal hemorrhage

## Abstract

Coronavirus disease 2019 (COVID-19) has been associated with thrombotic and endocrine complications, including adrenal insufficiency in the setting of adrenal hemorrhage. We present a patient diagnosed with antiphospholipid syndrome (APLS) in the setting of COVID-19 infection resulting in bilateral adrenal hemorrhage, subsequently leading to adrenal insufficiency. Acute adrenal hemorrhage is an underrecognized cause of decompensation, multisystem failure, and death in severe illness. Reports of adrenal insufficiency in the setting of COVID-19 infection revealed microscopic infarction, which can increase the risk of hemorrhage. Other mechanisms include severe hyperinflammatory response and cytokine storm leading to endothelial dysfunction, vascular injury, adrenal parenchymal damage, and hemorrhage. COVID-19 infection can be associated with coagulopathy and thromboembolic events and can lead to adrenal hemorrhage. Adrenal insufficiency is life-threatening and needs to be recognized promptly. There can be a latent phase between hemorrhagic events and adrenal failure; hence, close monitoring and timely intervention are important.

## Introduction

Coronavirus disease 2019 (COVID-19), caused by severe acute respiratory syndrome coronavirus 2 (SARS-CoV-2), is associated with proinflammatory and prothrombotic complications and abnormal coagulation parameters, including elevated D-dimer and prolonged prothrombin time. COVID-19 infection has been observed to affect various endocrine organs, including the adrenal glands. One proposed mechanism is the binding of the virus to angiotensin-converting enzyme 2 receptors present in endocrine glands [1]. Adrenal insufficiency has been noted as a possible early and late complication of COVID-19 infection. Proposed pathophysiological mechanisms include adrenal hemorrhage, adrenal infarction, ischemic necrosis, and adrenalitis [1].

Although COVID-19 has been associated with prothrombotic manifestations, there are few reports involving the adrenal gland and COVID-19 coagulopathy. Here, we present a case of a 50-year-old male who was diagnosed with antiphospholipid syndrome (APLS) in the setting of COVID-19 infection and was subsequently noted to have bilateral adrenal hemorrhage (BAH) leading to primary adrenal insufficiency with preserved mineralocorticoid function.

## Case presentation

A 50-year-old man with a past medical history of hypertension and gastroesophageal reflux was transferred to our institution for the management of intracerebral hemorrhages. A month prior to presentation, he tested positive for SARS-CoV-2 infection based on polymerase chain reaction (PCR) testing. At the time of diagnosis, his symptoms were nausea, vomiting, and generalized weakness. Over the next month, he developed myalgias, fevers, loss of appetite, and a weight loss of 45 lbs. These symptoms prompted his presentation to the emergency room, where he was noted to have an elevated heart rate of 130 beats per minute and an elevated respiratory rate of 24 cycles per minute. His blood pressure was 112/57 mm Hg and he was febrile to 101.4°F.

**Figure 1:**
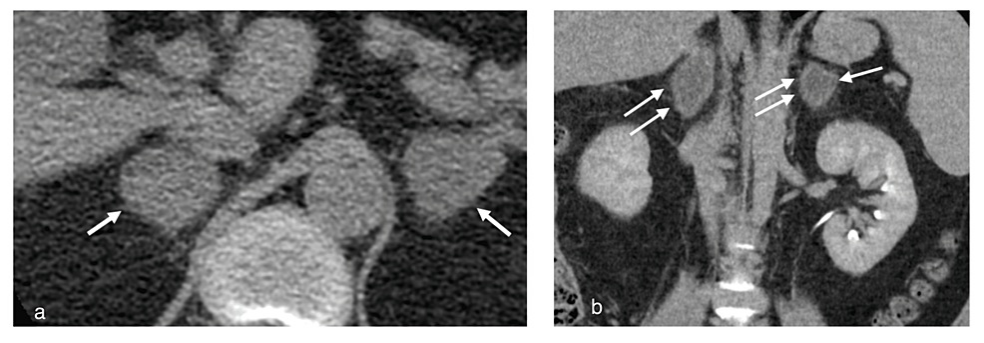
(a) Pre-contrast CT image showing bilateral adrenal masses (arrows). (b) Post-contrast images showing focal preservation of normal adrenal enhancement in the periphery (arrows)

His initial labs were notable for hyponatremia, elevated erythrocyte sedimentation rate and C-reactive protein levels, low hemoglobin, elevated D-dimer, elevated fibrinogen, prolonged prothrombin time (PT), and elevated international normalized ratio (INR) (Table 1). A repeat SARS-CoV-2 PCR test was negative. A chest CT angiography ruled out pulmonary embolism but noted indeterminate bilateral adrenal masses. A follow-up CT scan of his abdomen confirmed the presence of bilateral adrenal masses with low attenuation, measuring 3.9 x 2.5 cm on the right and 3.6 x 2.9 cm on the left, suggestive of adrenal hemorrhage/hematomas (Figure 1).

**Table 1:** Results of pertinent laboratory evaluation

Pertinent labs during hospital admission		
Lab	Results	Reference range
Sodium	128 mmol/L	136-145 mmol/L
Potassium	3.7 mmol/L	3.5-5.1 mmol/L
Glucose	118 mg/dL	70-100 mg/dL
Creatinine	1.1 mg/dL	0.6-1.1 mg/dL
C-reactive protein	9.2 mg/L	0-5 mg/L
Erythrocyte sedimentation rate	45 mm/hour	0-20 mm/hour
International normalized ratio	1.9	0.9-1.1
Prothrombin time	22 seconds	9.4-12.5 seconds
Activated partial thromboplastin time	313.3 seconds	25-36.5 seconds
Fibrinogen	745 mg/dL	180-400 mg/dL
D-dimer	1412 ng/mL	0-500 ng/mL
Lupus anticoagulant	Present	Not detected
Cardiolipin antibody IgG	81 GPL	14
Cardiolipin antibody IgM	63 MPL	12
Beta-2 glycoprotein I IgG antibody	101	20 SGU
Beta-2 glycoprotein I IgM antibody	10	20 SMU
Beta-2 glycoprotein I IgA antibody	>150	20 SAU

He was found to have elevated troponin I of 4.22 ng/mL and was started on a heparin drip. Twelve hours after the heparin drip was initiated, his hemoglobin dropped from 9.7 to 5.7 g/dL. The heparin drip was stopped, and he received blood transfusions. He then developed altered mental status, decreased strength in his lower extremities, and absent reflexes. Guillain-Barré syndrome was considered; however, an MRI of the brain revealed multiple acute hemorrhagic infarcts. Further, the work-up revealed a strongly positive phospholipid antibody panel. His presentation was felt to be most consistent with APLS, inducing a hypercoagulable state, possibly triggered by COVID-19 infection. He was started on a prednisone taper along with hydroxychloroquine and anticoagulation with warfarin.

About one week after the initial scan, a repeat CT of the abdomen with multiphasic adrenal protocol demonstrated bilateral intermediate density non-enhancing adrenal masses, most suggestive of BAH. The right-sided mass measured 30 Hounsfield units (HU) on the non-contrast phase, 28 HU on the portal venous phase, and 30 HU on the 15-minute delayed phase. The left-sided mass measured 24 HU on the non-contrast phase, 22 HU on the portal venous phase, and 21 HU on the 15-minute delayed phase. Morning cortisol prior to initiating prednisone was 9.3 ug/dL.

The diagnosis of APLS was confirmed with a repeat phospholipid panel three months after the initial labs. He was continued on hydroxychloroquine, and prednisone was tapered down to 5 mg daily over the course of seven months. At this point, he was referred to the endocrinology clinic for assessment of his adrenal function. He was hypertensive with a blood pressure of 144/96 mmHg. Physical exam was notable for mild skin hyperpigmentation and normal body hair distribution. His 8 am cortisol level after holding prednisone for 24 hours was 2.2 ug/dL and adrenocorticotropic hormone (ACTH) was 400 pg/mL, suggesting primary adrenal insufficiency, confirmed with cosyntropin stimulation testing (Table 2).

**Table 2:** Subsequent outpatient endocrine testing LC-MS: liquid chromatography-mass spectrometry.

Labs	Baseline	30 minutes	60 minutes	Reference range
Adrenocorticotropic hormone	400 pg/mL	-	-	6-50 pg/mL
Cortisol (8 am)	2.5 ug/dL	2.6 ug/dL	2.6 ug/dL	2-20 ug/dL (7-10 AM: 6.0-18.4 ug/dL)
Aldosterone (LC-MS)	8 ng/dL	10 ng/dL	8 ng/dL	< or = 28 ng/dL (upright 8-10 am)
Renin	3.3 ng/mL/hr	-	-	0.25-5.82 ng/mL/hr
21-hydroxylase antibody	Negative	-	-	
Thyroid-stimulating hormone	1.9 uIU/mL	-	-	0.27-4.2 uIU/mL

**Figure 2:**
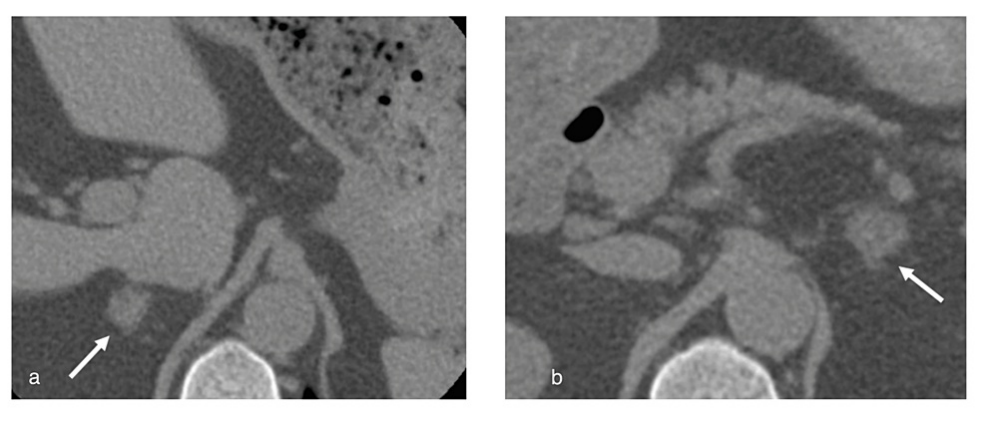
Adrenal CT showing interval decrease in the size of adrenal hemorrhage bilaterally compared to the initial presentation

A repeat CT scan eight months after the initial presentation was notable for an interval decrease in adrenal hemorrhage with only a trace amount of normal adrenal tissue remaining (Figure 2). He was continued on prednisone 5 mg. He remained mildly hypertensive, with normal potassium and sodium levels, robust baseline aldosterone level, and normal renin level, so fludrocortisone was not initiated.

## Discussion

Acute adrenal hemorrhage is an underrecognized cause of decompensation, multisystem failure, and death in severe illness [2]. Adrenal hemorrhage associated with sepsis is known as Waterhouse-Friderichsen syndrome. While originally it was associated with *Neisseria meningitidis*, various other organisms have been implicated, including viruses such as cytomegalovirus and parvovirus B19 [3]. Some case reports have identified patients with BAH and adrenal insufficiency in the setting of COVID-19 infection. Other case reports and case series of COVID-19 and adrenal insufficiency revealed microscopic infarction, which can increase the risk of hemorrhage [4]. Studies have shown that the SARS-CoV-2 virus enters the cells and binds angiotensin-converting enzyme receptors, resulting in coagulation dysregulation and thrombosis. Other possible mechanisms include severe hyperinflammatory response and cytokine storm leading to endothelial dysfunction, vascular injury, and adrenal parenchymal damage and hemorrhage. Further, the vascular anatomy of the adrenal glands, with three arteries and one vein, predisposes them to hemorrhagic necrosis [5].

APLS includes a clinical event (thrombosis, pregnancy loss, etc.) and persistently positive antiphospholipid antibodies. BAH in the context of a hypercoagulable state such as APLS or heparin-induced thrombocytopenia (HIT) has been described [6,7]. One such study described 16 patients who were found to have adrenal insufficiency in the setting of APLS [6]. Similar to our case, CT scans revealed diffusely enlarged adrenal glands or bilateral masses. Synacthen stimulation testing in eight out of 10 patients revealed no significant rise in cortisol, and aldosterone levels were low or undetectable in four out of six patients tested.

Interestingly, our patient did not develop mineralocorticoid deficiency and labs revealed normal aldosterone levels. A small percentage of patients with primary adrenal insufficiency may not need mineralocorticoid replacement. There has been only one other case report of BAH in the setting of COVID-19 where the patient had isolated glucocorticoid insufficiency [8]. A case report describing the evolution of adrenal dysfunction after bilateral infarction revealed the development of mineralocorticoid deficiency seven months after initial presentation, suggesting possible delayed presentation [9]. Our patient did not develop mineralocorticoid insufficiency even after 28 months from the initial presentation.

There has been a single confirmed report of adrenal hemorrhage in the setting of COVID-19 and newly diagnosed APLS before ours [10]. In that case, the patient had a history of multiple prior abortions, presented with COVID-19, and was found to have a BAH, adrenal insufficiency, and a positive antiphospholipid antibody profile. She required treatment with glucocorticoid and mineralocorticoid replacement. This differs from our case where the patient was started on glucocorticoids for a different condition (autoimmune hemolytic anemia), later found to have adrenal insufficiency on follow-up testing several months later, and did not require mineralocorticoids.

Another recent review discussed COVID-19-related adrenal hemorrhage, both infection-related and vaccine-related. Of the 18 cases included in the study, seven patients had infection-related adrenal hemorrhage. The median time from a positive COVID-19 test to the development of adrenal hemorrhage was eight days (range = 1-30) [11].

A total of 16 patients (12 case reports, four from case series) have been reported so far with confirmed BAH or infarction in the setting of COVID-19 infection (Table 3). Treatment with steroids was needed for 14 of them (one with no reported treatment). Eight of these 16 patients had BAH and two of these patients were newly diagnosed with APLS and two had evidence of only glucocorticoid insufficiency, including ours.

**Table 3:** Prior published reports of bilateral adrenal hemorrhage in the setting of COVID-19 infection ACTH: adrenocorticotropic hormone; BAH: bilateral adrenal hemorrhage; NM: value was not measured; APLS: antiphospholipid syndrome; Hydrocort: hydrocortisone; Fludrocort: fludrocortisone.

­	Age, sex	Baseline cortisol (μg/dL)	Aldosterone (ng/dL)	ACTH	Significant medical conditions	Imaging	Anticoagulant exposure preceding adrenal hemorrhage	Treatment
Case reports								
Present case	54 M	2.5	8	400 pg/mL (ref: 6-50)	+ APLS, new diagnosis + autoimmune hemolytic anemia	BAH	+	Prednisone
Alvarez-Troncoso et al. (2020) [8]	70 M	2.1	Normal (No value reported)	NM	+ Mild psoriasis	Acute BAH. Enlargement and blurring (loss of Y shape) of both adrenals		Hydrocort
Frankel et al. (2020) [10]	66 F	<1	NM	207 pmol/L (ref: 1.6-13.9)	+ APLS	BAH. Thickened and enlarged adrenals, haziness of peri-adrenal fat. Rounded right adrenal and V-shaped left adrenal. Lack of contrast enhancement		Prednisone, fludrocort
Miranda et al. (2021) [12]	47 M	NM	NM	NM	NM	Subacute BAH	+	None
Sreedharan et al. (2022) [13]	NM	NM	NM	NM	NM	BAH	NM	NM
Elkhouly et al. (2021) [14]	50 M	26	NM	NM	+ Hypertension, right adrenal adenoma	BAH	+	Hydrocort (Deceased in the hospital)
Jaiswal and Schulman‐ Rosenbaum (2021) [15]	71 F	10	NM	3 pg/mL (While on steroid replacement)	+ Hypertension, hyperlipidemia, type 2 diabetes mellitus	BAH	-	Hydrocort Fludrocort
Zilberman et al. (2023) [16]	89 M	8.4	NM	NM	+ Prostate cancer s/p brachytherapy	BAH	-	Hydrocort
Kumar et al. (2020) [17]	70 F	Normal (No value reported)	NM	Normal (No value reported)	+ Hypertension	Bilateral adrenal non-hemorrhagic infarction. Ill-defined contours and retroperitoneal fat stranding	-	Hydrocort
Machado et al. (2022) [18]	46 F	<1.0	<3 ng/dL	807 pg/mL	+ APLS, new diagnosis	Bilateral adrenal non-hemorrhagic infarction. Left adrenal vein thrombosis	-	Hydrocort Fludrocort
Haider et al. (2021) [19]	71 M	Low (No value reported)	NM	NM	NM	Bilateral adrenal non-hemorrhagic infarction. Fat stranding around both adrenals	+	Hydrocort Fludrocort
Asano et al. (2021) [20]	76 F	29.7	NM	176.6 pg/mL	+ Sjogren’s syndrome	Bilateral adrenal non-hemorrhagic infarction	+	Hydrocort
Case series: Leyendecker et al. (2021) [4]								
A total of 51­ patients with adrenal infarction on imaging. A total of 45 patients had bilateral adrenal infarction, of which four patients (8%) had adrenal insufficiency. Imaging findings in all 45 patients were consistent with bilateral adrenal infarction. Additional clinical history for these patients is unavailable.								

## Conclusions

In situations of increased stress, increased adrenal blood flow, hypotension, and/or coagulopathy, the adrenal gland is predisposed to hemorrhagic necrosis. Our patient presented with BAH, with likely contributions from COVID-19 infection and newly diagnosed APLS. Our case highlights the association between severe COVID-19 infection and potential adrenal complications, especially in the setting of coagulopathic disorder or therapeutic anticoagulation treatment. Patients with antiphospholipid antibodies in the setting of COVID-19 infection and who are treated with therapeutic anticoagulation are at higher risk of developing adrenal hemorrhage and subsequently primary adrenal insufficiency. Spontaneous BAH is rare and potentially fatal if not diagnosed promptly. These patients often present with nonspecific symptoms, and are now frequently initiated on dexamethasone inpatient, potentially complicating and delaying the diagnosis of adrenal insufficiency. Clinicians should be aware of the association between antiphospholipid syndrome and adrenal hemorrhage with the potential to develop adrenal failure, which is amenable to treatment with corticosteroids.
